# Survey of physician and pharmacist steward perceptions of their antibiotic stewardship programs

**DOI:** 10.1017/ash.2021.219

**Published:** 2021-11-12

**Authors:** Shana A. B. Burrowes, Mari-Lynn Drainoni, Maria Tjilos, Jorie M. Butler, Laura J. Damschroder, Matthew Bidwell Goetz, Karl Madaras-Kelly, Caitlin M. Reardon, Matthew H. Samore, Jincheng Shen, Edward Stenehjem, Yue Zhang, Tamar F. Barlam

**Affiliations:** 1 Section of Infectious Diseases, Department of Medicine, School of Medicine, Boston University, Boston, Massachusetts; 2 Department of Health Law, Policy and Management, School of Public Health, Boston University, Boston, Massachusetts; 3 Evans Center for Implementation and Improvement Sciences, Department of Medicine, School of Medicine, Boston University, Boston, Massachusetts; 4 Department of Community Health Sciences, School of Public Health, Boston University, Boston, Massachusetts; 5 Division of Geriatrics, Department of Internal Medicine, University of Utah, Salt Lake City, Utah; 6 Geriatric Education and Clinical Center and IDEAS Center of Innovation, Veterans’ Affairs (VA) Salt Lake City Health Care System, Salt Lake City, Utah; 7 VA Center for Clinical Management Research, Department of Veterans’ Affairs, Ann Arbor, Michigan; 8 VA Greater Los Angeles Healthcare System, Los Angeles, California; 9 David Geffen School of Medicine, UCLA, Los Angeles, California; 10 Boise VA Medical Center, Boise, Idaho; 11 College of Pharmacy, Idaho State University, Meridian, Idaho; 12 IDEAS Center of Innovation, VA Salt Lake City Health Care System, Salt Lake City, Utah; 13 Division of Epidemiology, Department of Internal Medicine, School of Medicine, University of Utah, Salt Lake City, Utah; 14 Department of Population Health Sciences, University of Utah, Salt Lake City, Utah; 15 Office of Patient Experience, Intermountain Healthcare, Salt Lake City, Utah

**Keywords:** antbiotic stewardship, pharmacist stewards, volunteer stewards

## Abstract

**Objective:**

To examine how individual steward characteristics (eg, steward role, sex, and specialized training) are associated with their views of antimicrobial stewardship program (ASP) implementation at their institution.

**Design:**

Descriptive survey from a mixed-methods study.

**Setting:**

Two large national healthcare systems; the Veterans’ Health Administration (VA) (n = 134 hospitals) and Intermountain Healthcare (IHC; n = 20 hospitals).

**Participants:**

We sent the survey to 329 antibiotic stewards serving in 154 hospitals; 152 were physicians and 177 were pharmacists. In total, 118 pharmacists and 64 physicians from 126 hospitals responded.

**Methods:**

The survey was grounded in constructs of the Consolidated Framework for Implementation Research, and it assessed stewards’ views on the development and implementation of antibiotic stewardship programs (ASPs) at their institutions We then examined differences in stewards’ views by demographic factors.

**Results:**

Regardless of individual factors, stewards agreed that the ASP added value to their institution and was advantageous to patient care. Stewards also reported high levels of collegiality and self-efficacy. Stewards who had specialized training or those volunteered for the role were less likely to think that the ASP was implemented due to a mandate. Similarly volunteers and those with specialized training felt that they had authority in the antibiotic decisions made in their facility.

**Conclusions:**

Given the importance of ASPs, it may be beneficial for healthcare institutions to recruit and train individuals with a true interest in stewardship.

Infections from antibiotic-resistant bacteria result in increased patient morbidity, mortality, and healthcare-associated costs.^1^ One driving factor for growing resistance is the inappropriate use of antibiotics; ∼50% of antibiotic use in hospitals is inappropriate.^1^ Antibiotic stewardship programs (ASPs) have been mandated across healthcare settings in response to the growing public health crisis of antibiotic resistance.^2^ Effective ASPs reduce length of stay and improve the judicious use of antibiotics, patient satisfaction, and patient outcomes.^1,3^ Although the Centers for Disease Control and Prevention (CDC) has recommended several core elements necessary for effective ASPs, institutions have flexibility in the way those core elements are implemented,^4,5^ which can lead to varying results in the metrics of ASP success such as antibiotic use or cost savings.^6^

ASP implementation has multiple facilitators and challenges in hospital settings, including facility-level factors and provider inter- and intrapersonal factors.^1,7^ Several studies have identified inadequate financial resources to pay for antibiotic stewardship staff time as a barrier to ASP implementation.^1,5,8^ Lack of education and training regarding appropriate antimicrobial use, as well as poor communication from leadership to hospital staff, are further barriers.^1,7^ Physician stewards are generally trained in infectious diseases (ID) and have expertise in treating antimicrobial infections and knowledge of resistance and pharmacokinetics.^3^ Pharmacy stewards receive training focused on skills to evaluate and design treatment plans for complex ID scenarios; they also compare and contrast antimicrobial agents to help develop ASP strategies.^9^ Collegial relationships between the stewardship team and other staff facilitate ASP implementation.^5^ Our team recently showed that the physician–pharmacist steward relationship and steward engagement with other providers was viewed as critical to the success of stewardship programs.^10^

Although prior research has examined barriers and facilitators to ASPs, the variation in these factors based on individual characteristics of stewards has not been fully explored. We describe a survey of pharmacist and physician stewards across 2 large healthcare systems to examine how individual steward characteristics (eg, steward role, gender, specialized training) are associated with their views of ASP implementation at their institution. Assessing these views is important for identifying characteristics of potential stewards who might be successful in the role.

## Methods

### Study design and participants

This research represents a subanalysis of a larger mixed-methods study examining the implementation of inpatient ASPs in 2 large national healthcare systems, the Veterans’ Health Administration (VA; n = 134) and Intermountain Healthcare (IHC; n = 20). We developed a survey grounded in constructs of the Consolidated Framework for Implementation Research (CFIR)^11^ to understand stewards’ perspectives of ASPs and their implementation. The CFIR framework consists of 39 constructs within 5 primary domains: intervention characteristics, outer setting, inner setting, characteristics of the individual, and the implementation process. The final survey created for this study comprised 62 questions and covered multiple topics: stewards’ views on ASPs in general, the development of the ASP within their institution, perceptions of staff and leadership engagement with the ASP, and stewards’ opinions of their own self-efficacy. We mapped each question to the relevant CFIR construct and domain (Supplementary Table 1).

**Table 1.** Demographic Characteristics of Antibiotics Stewards at Veterans’ Affairs and Intermountain Health System Hospitals (n = 182)

Variable	Total Sample, No. (%)^a^	Physicians, No. (%)^b^	Pharmacists, No. (%)^b^
Survey participants	182	64 (35.16)	118 (64.84)
Intermountain Hospital	27 (14.84)	11 (40.74)	16 (59.26)
VA hospital	155 (85.16)	53 (34.19)	102 (65.81)
**Sex**			
Female	92 (50.55)	25 (27.17)	67 (72.83)
Male	90 (49.45)	39 (43.33)	51 (56.67)
**Specialized training**^c^			
Yes	132 (73.74)	55 (41.67)	77 (58.33)
No	47 (26.26)	7 (14.89)	40 (85.11)
**Steward selection**^d^			
Volunteered	92 (51.69)	33 (35.87)	59 (64.13)
Expressed interest but assigned role	35 (19.66)	11 (31.43)	24 (68.57)
Assigned role	51 (28.65)	17 (33.33)	34 (66.67)
**Involvement in stewardship program**^e^			
Primary leader	40 (22.22)	17 (42.5)	23 (57.5)
Co-lead	107 (59.44)	35 (32.71)	72 (67.29)
Do a lot of the work but I’m not in charge	27 (15)	6 (22.22)	21 (77.78)
Other	6 (3.33)	4(66.67)	2 (33.33)
Time in facility median y ±IQR	7.5±10	7±12	8±9
Time licensed, median y ±IQR	12±15	15±12	11±15
Interest value median ± IQR	97±23	86±30.5	99±18

Note. IQR, interquartile range.

a Column percent.

b Row percent.

c Specialized training; 3 responses missing.

d Steward selection; 4 responses missing

e Involvement; 2 responses missing.

The electronic survey was conducted with physician and pharmacist stewards in the 2 healthcare systems. In addition to the CFIR construct questions, 10 questions were used to collect demographic data regarding the following factors: sex, whether the steward was a physician or pharmacist, stewards’ perception of their role in the ASP (primary leader, coleader, do a lot of the work but not in charge, other), specialized training (in infectious diseases or antibiotic management), level of interest in antibiotic stewardship when they first started their stewardship position (measured on a scale from 0 to 100) and how they became involved in the ASP (ie, volunteered, expressed interest but was assigned, or was assigned). We sent the survey to 329 antibiotic stewards from 154 hospitals; 152 were physicians and 177 were pharmacists. We received responses from 126 hospitals (109 VA hospitals and 17 IHC hospitals), which represented an 81.8% hospital response rate with 182 (55.3%) of 329 steward responses. The Boston University Medical Center Institutional Review Board approved the subanalysis of steward surveys.

### Data analysis

Exploratory analyses examined each hospital system (VA vs IHC) separately, but due to the comparatively small number of IHC hospitals included, final statistical analyses combined IHC and VA responses. Survey responses were recorded on a 6-point Likert scale: strongly agree, agree, neither agree or disagree, disagree, strongly disagree, don’t know. Univariate analyses wre used to examine the distribution of responses across all CFIR constructs; bivariate analyses were used to examine differences in responses across survey topics and demographic factors. We combined the responses of strongly agree and agree into 1 category, and we similarly combined responses of strongly disagree and disagree. The χ^2^ and Fisher exact tests were used for categorical variables and 2-sample *t* tests, the Wilcoxon and Kruskal-Wallis tests were used for continuous variables. Testing was 2-sided and an alpha level of .05 was considered significant; however, due to small cell sizes, *P* values are not reported. All statistical analyses were conducted using SAS version 9.4 software (SAS Institute, Cary, NC).

## Results

### Overview of survey participants

Of the 182 respondents, 118 (65%) were pharmacists and 64 (35%) were physicians. Respondents were primarily female (n = 92, 51%), and most respondents had specialized training (n = 132, 73.7%). They had been licensed for a median of 12 years. Respondents had worked at their institution for a median of 7.5 years (Table 1). Female stewards were more likely to be pharmacists and physician stewards more often had specialized training. Of the stewards surveyed, 92 (52%) volunteered for the position, 51 (29%) had been assigned to the steward role, and 35 (20%) expressed interest in the role and were assigned.

### Stewards’ views of the ASP

The results from all survey questions are displayed in Supplementary Figures 1a–d. Regardless of individual factors, almost all stewards agreed that the ASP added value to their institution (n = 174, 95.6%) and was advantageous to patients (n = 175, 96.7%). Stewards reported high self-efficacy, with 87% stating that they had the skills to function effectively in their role. Self-efficacy was similar between pharmacists and physicians and did not differ by sex, specialized training, or whether the steward viewed themselves as a leader in the ASP. A high level of collegiality was reported across all surveyed stewards regardless of their individual characteristics. Stewards agreed that they worked well on interdisciplinary teams (n = 172, 95%) and with individual clinicians (n = 176, 96.7%). When asked about the development and implementation of the ASP within their institution, most stewards agreed that there was a champion on the clinical staff who actively promoted the ASP (n = 140, 76.9%) and that clinical leadership gave them the authority to enforce stewardship activities (n = 128, 71.1%). However, fewer stewards (n = 79, 43.4%) agreed that hospital leadership provided the adequate resources needed to establish the ASP.

**Fig. 1.**
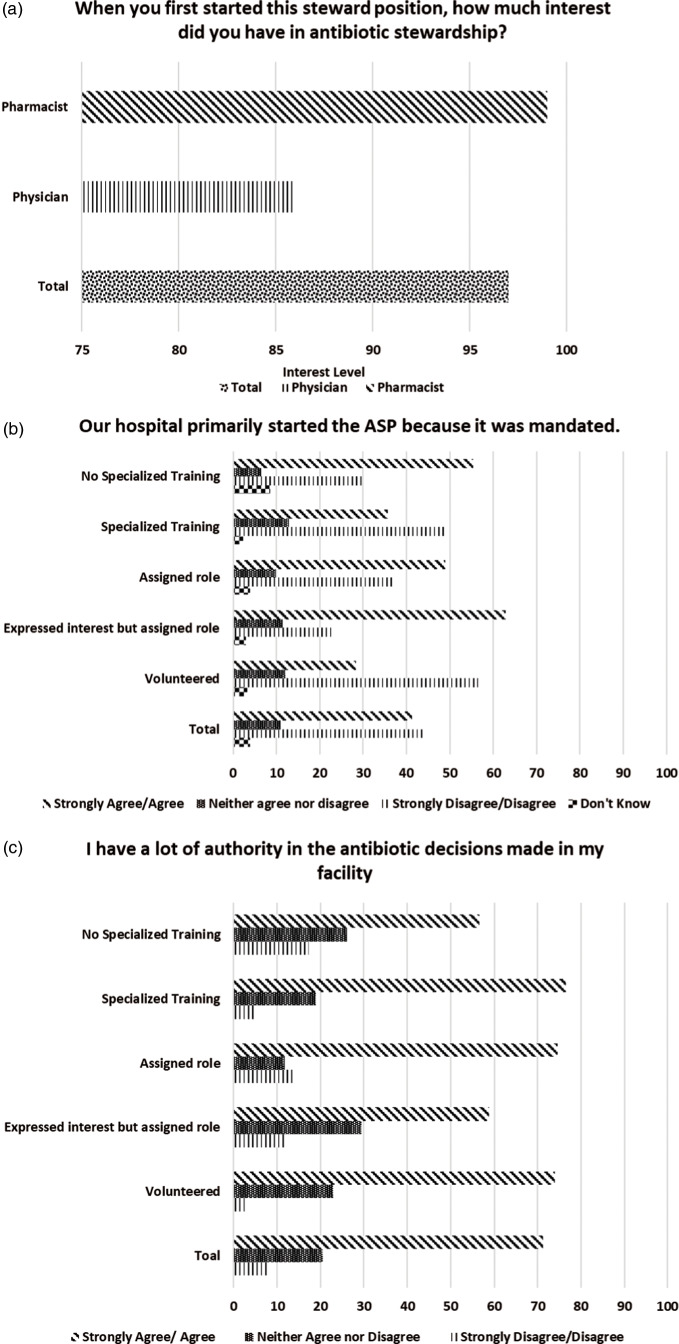
Stewards’ views on antibiotic stewardship programs a by individual steward characteristics. Bar chart to show the breakdown of stewards’ responses by individual characteristics. The specific survey question is stated at the top of each figure. Part (a) shows differences between physicians and pharmacists. Parts (b) and (c) show differences by volunteer status and specialized training.

In some instances, views on the ASP differed based on stewards’ individual demographic factors. Although overall interest in the ASP was high (median ± IQR, 97±23), pharmacists gave this question a higher value (median ± IQR, 99±18) than physicians (median ± IQR, 86±30.5) (Fig. 1a). Stewards were undecided about whether the ASP was initially implemented due an internal push from hospital leadership (38.9% agreed and 37.2% disagreed) or due to a mandate (41.2% agreed and 44% disagreed). These responses were influenced by whether they had volunteered for the stewardship position (Fig. 1b). Stewards who volunteered (56.2%) more often disagreed that the ASP was started due to an external mandate. These findings contrast with the responses of those who expressed interest but were assigned to the role (22.9%) and with those who were simply assigned (37.3%). Views on the initial implementation of the ASP also differed by whether the steward had specialized training. Stewards with specialized training (49.2%) more often disagreed that the ASP was started due to mandate compared to those without this training (29.8%). Additionally stewards who disagreed that the ASP was due to an internal push had been licensed for a median of 16 years compared to 11 years in those who agreed. Furthermore, when asked whether clinical leadership endorsed the program in visible ways, 50% of physicians agreed compared to 72.6% of pharmacists.

Stewards had different views when asked whether they had a great deal of authority in the antibiotic decisions made in their facility (Fig. 1c). Among those with specialized training, 76.5% agreed they had that authority, compared to 56.5% of those without specialized training. In terms of volunteer status, 73.9% of stewards who volunteered and 74.5% of those who were assigned to the role agreed that they had authority in antibiotic decisions. Conversely, only 58.8% of those who expressed interest but were assigned agreed that this was the case. Finally, stewards who agreed that staff had a sense of personal responsibility for improving patient care and outcomes and that staff were receptive to changes in the clinical process had been licensed for a longer period of time than those who disagreed.

## Discussion

We have reported physician and pharmacist stewards’ views on the development and implementation of ASPs at their institutions. Generally, individual characteristics do not affect the majority of those views. Although we thought that gender bias in medicine may affect female stewards’ self-efficacy and perception of their authority, this was not the case. Stewards reported high interest in antibiotic stewardship; they often volunteered for the role and stated that the ASP was advantageous to both the institution and patient care. Physician and pharmacist stewards were engaged in their programs, felt strongly that there was buy-in from clinical staff, and felt that the clinical leadership provided stewards with the authority to enforce the ASP policies.

The commitment of hospital leadership to ASPs is a core element identified by the CDC and encompasses financial, human, and technological resources.^12^ In our study, stewards, regardless of individual characteristics, did not think that hospital leadership provided sufficient support in the form of protected time and resources to do the job. This finding is consistent with a qualitative study of pharmacists’ perspectives that found pharmacist champions and stewards in one healthcare system identified inadequate time for stewardship activities as a barrier to successful ASP implementation.^1^ In another study, a survey of ASP physicians and pharmacists from 21 academic medical centers revealed that in addition to a lack of dedicated time for stewardship, insufficient personnel, such as the absence of a full-time ID physician, resulted in delayed uptake of pharmacists ASP recommendations.^5^

Providing support to time-strapped ASP staff can be achieved in several ways. Access to computerized decision-support tools reduces the amount of time stewards need to do their job.^1^ In institutions with financial and human resource constraints, hospital leadership can provide access to external software if they are unavailable locally and/or if IT personnel are shared across institutions to support technological efforts in the ASP.^1^ If there is an absence of on-site ID expertise, leadership can also employ a telehealth approach in which ID consultations and stewardship support can be provided remotely.^1^Although the stewards in our study believed that dedicated financial support and protected time for their ASP were insufficient, this belief did not affect their views regarding their ability to do their job. Stewards reported high self-efficacy, stating that not only did they have the skills to do the job but also the authority to enforce ASP guidelines. Moreover, this level of self-efficacy was consistently reported across stewards regardless of sex, leadership role, specialized training, or whether the steward was a pharmacist or a physician. Our findings add to the growing literature supporting the expanding leadership role of pharmacists within ASPs and their strength in this role. For example, in France, where stewardship programs are led by ID physicians, a multicenter study of 88 hospitals reported that sites with pharmacist stewards, referred to as antibiotic advisors, had less antibiotic consumption than those with nonpharmacist advisors.^13^ This finding is consistent with those of Doernberg et al^8^ in 2018, which showed that increasing pharmacist FTE was associated with significant improvement in ASP effectiveness, whereas increasing physician FTE did not result in significant improvement.

Although research on ASPs often focuses on the importance of physical and financial resources,^3^ other components are crucial to success. In our study, stewards exhibited personal ownership of the program through their high level of volunteerism and specialized training in antibiotic education or infectious diseases. In addition, volunteers and those with specialized training were less likely to think that the ASP was mandated. This finding suggests that the stewards in our survey had a high interest in stewardship and would have been champions for ASP activities regardless of directives. The importance of personal investment has been highlighted in other healthcare settings. In a study conducted across three accountable care organizations, motivation for behavior change went beyond financial incentives; instead, factors related to a provider’s personal motivation, such as mastery of skill and a feeling of social purpose, were much more important.^14^ Additionally, our previous qualitative work with stewards in this project details similar motivations. Stewards not only welcomed learning new skills but also enjoyed their roles and felt that their job was important in improving clinical care.^10^

This study had several limitations. Although we recruited participants from 154 hospitals, they represented only 2 health systems; thus, our findings may not be generalizable to other healthcare institutions. Additionally, we combined responses across systems and this may have obscured differences between the VA and IHC sites. However, given that there was often general agreement, this is unlikely. Stewards may have self-selected to respond to the survey, and those with less interest in the ASP may not have responded; therefore, the results may not reflect the views of all stewards; however, the high survey response rate (81.8%) makes this less likely. Even with these limitations, our findings highlight the fact that that interpersonal factors are important in the development and sustainability of ASPs.

In conclusion, stewards generally had positive views toward the ASPs in their institutions. Those who volunteered for their role or had specialized training more often viewed the ASP in a positive light. Our findings suggest that, though insufficient financial support and limited staff can be barriers to a successful ASP, the personal investment of stewards is critical to success. These findings are timely and indicate that in a new climate in which ASPs are required, hospitals might see the full benefits of their programs if they recruit and provide training to individuals with a true interest in stewardship.

## Data Availability

Data are available upon request.

## Supplementary material

For supplementary material accompanying this paper visit https://doi.org/10.1017/ash.2021.219. click here to view supplementary material
