# Supplementary material for: Survey of physician and pharmacist steward perceptions of their antibiotic stewardship programs
Source: Antimicrob Steward Healthc Epidemiol. 2021 Nov 12;1(1):e48. doi: 10.1017/ash.2021.219 (PMC9495632; doi:10.1017/ash.2021.219)
Supplement: Supplementary file 1 [file S2732494X21002199sup001.docx]

**Supplementary Table 1: Antibiotic Stewardship Survey Linked to CFIR Domains and Constructs**

| **Question Theme** | **Question** | **CFIR Construct** | **CFIR Domain** |
| --- | --- | --- | --- |
| **About ASPs** | Academic literature confirms that ASPs are an effective way to improve antibiotic prescribing | **Evidence Strength & Quality** | **Intervention Characteristics** |
|  | Clinical experience confirms that ASPs are an effective way to improve antibiotic prescribing. | **Evidence Strength & Quality** | **Intervention Characteristics** |
|  | ASPs add value to our organization. | **Relative Advantage** | **Intervention Characteristics** |
|  | ASPs appear to have **more advantages** than disadvantages for our patients. | **Relative Advantage** | **Intervention Characteristics** |
| **ASPs in this setting** | Our hospital primarily started the ASP because it was mandated. | **Leadership Engagement** | **Inner Setting** |
|  | There was an internal push from hospital leadership to establish an ASP. | **Leadership Engagement** | **Inner Setting** |
|  | There was an ASP-champion on the clinical staff who actively promoted the implementation of the ASP. | **Leadership Engagement** | **Inner Setting** |
|  | Knowing that other peer hospitals were instituting an ASP was a factor in deciding to implement one here. | **Peer Pressure** | **Outer Setting** |
|  | Talking to other organizations with ASPs helped us develop our ASP. | **Cosmopolitanism** | **Outer Setting** |
|  | Hospital leadership was involved in the initial development of the ASP. | **Readiness for Implementation: Leadership Engagement** | **Inner Setting** |
|  | Hospital leadership provided adequate resources to establish the ASP. | **Readiness for Implementation: Leadership Engagement** | **Inner Setting** |
|  | When the ASP was initially developed, roles and responsibilities were clearly identified. | **Planning** | **Process** |
|  | A realistic time schedule was developed for implementation of the ASP when the program was initially implemented. | **Planning** | **Process** |
|  | The original plan for implementing the ASP acknowledged clinicians’ input and opinions. | **Planning** | **Process** |
|  | The original plan for implementing the ASP was unnecessarily complex. | **Planning** | **Process** |
|  | This ASP had the support of the key opinion leaders in the hospital. | **Engaging: Opinion Leaders** | **Process** |
| **Leadership’s perspective on Local ASP** | Hospital leadership sets a high priority on the success of the ASP. | **Relative Priority** | **Inner Setting** |
|  | Clinical leadership has endorsed the project in visible ways. | **Leadership Engagement** | **Inner Setting** |
|  | Clinical leadership gives the antibiotic steward the authority to enforce the ASP policies. | **Leadership Engagement** | **Inner Setting** |
|  | The Antibiotic steward(s) has protected time to implement the ASP. | **Leadership Engagement** | **Inner Setting** |
|  | The ASP has been easily integrated into the Electronic Health Record (EHR) system. | **Compatibility** | **Inner Setting** |
|  | The EHR provides helpful reports about antibiotic use within the hospital. | **Access to knowledge & info** | **Inner Setting** |
|  | The ASP has considerable visibility within the hospital system. | **Engaging** | **Process** |
|  | Hospital leadership receives regular feedback on progress of ASP activities and resource needs. | **Reflecting & Evaluating** | **Process** |
| **Staff’s perspective on local ASP** | At my hospital… |  |  |
|  | Clinicians and hospital staff have received enough education and training on the ASP. | **Access to Knowledge & Info** | **Inner Setting** |
|  | The ASP is integrated into new provider training. | **Access to Knowledge & Info** | **Inner Setting** |
|  | The staff was receptive to the ASP. | **Knowledge & Beliefs about the Intervention** | **Characteristics of the Individual** |
|  | Nursing and other support staff understand the importance of the ASP policies. | **Knowledge & Beliefs about the Intervention** | **Characteristics of the Individual** |
|  | ASP policies put a heavy burden on the nursing staff. | **Knowledge & Beliefs about the Intervention** | **Characteristics of the Individual** |
|  | Nurses are actively engaged in the ASP activities. | **Executing** | **Process** |
|  | Clinical Pharmacists who provide patient care on the inpatient wards understand the importance of the ASP policies. | **Knowledge & Beliefs about the Intervention** | **Characteristics of the Individual** |
|  | ASP policies put a heavy burden on Clinical Pharmacists who provide patient care on inpatient wards. | **Knowledge & Beliefs about the Intervention** | **Characteristics of the Individual** |
|  | Clinical Pharmacists who provide patient care on the inpatient wards are actively engaged in ASP activities. | **Executing** | **Process** |
|  | Clinicians do not like the ASP because they feel it limits their ability to treat patients the way they think is best. | **Knowledge & Beliefs about the Intervention** | **Characteristics of the Individual** |
|  | Clinicians think the ASP delays antibiotic therapy too much. | **Knowledge & Beliefs about the Intervention** | **Characteristics of the Individual** |
|  | Clinicians think the ASP restricts too many antibiotics. | **Knowledge & Beliefs about the Intervention** | **Characteristics of the Individual** |
|  | The ASP involves too many steps for clinicians to adhere to in prescribing antibiotics. | **Knowledge & Beliefs about the Intervention** | **Characteristics of the Individual** |
|  | The ASP is strongly supported within the organization. | **Engaging** | **Process** |
|  | Feedback from clinicians related to proposed and implemented changes of the ASP is collected regularly. | **Reflecting and Evaluating** | **Process** |
|  | Clinicians at my facility view ASPs as one of the more important interventions the hospital can adopt compared to other interventions. | **Implementation Climate: Compatibility** | **Inner Setting** |
| **Opinions of the Steward** | As an antibiotic steward… |  |  |
|  | I work well with the interdisciplinary medical teams. | **Engaging: Formally Appointed Internal Implementation Leaders** | **Process** |
|  | I work well with individual clinicians. | **Engaging: Formally Appointed Internal Implementation Leaders** | **Process** |
|  | I have a lot of authority in the antibiotic decisions made in my facility. | **Self-Efficacy** | **Characteristics of the Individual** |
|  | I provide options to clinicians in antibiotic decisions in my facility. | **Self-Efficacy** | **Characteristics of the Individual** |
|  | I accept responsibility for the outcomes of this program. | **Self-Efficacy** | **Characteristics of the Individual** |
|  | I feel like I can effect change in my setting. | **Self-Efficacy** | **Characteristics of the Individual** |
|  | I feel like I have the skills to function effectively in my role. | **Self-Efficacy** | **Characteristics of the Individual** |
|  | I am able to carry out the mission of the ASP in my hospital. | **Self-Efficacy** | **Characteristics of the Individual** |
|  | I am empowered to continue to improve the ASP. | **Self-Efficacy** | **Characteristics of the Individual** |
|  | I am invested in the success of the ASP. | **Self-Efficacy** | **Characteristics of the Individual** |
|  | It is important to me that my hospital does not have substantially higher antibiotic use compared to other hospitals. | **Individual Identification with Organization** | **Characteristics of the Individual** |
| **About the Hospital** | In this hospital, clinical staff’s opinions are solicited regarding decisions about patient care. | **Networks & Communication** | **Inner Setting** |
|  | In this hospital, clinical staff’s opinions are solicited regarding innovative, new programs. | **Learning Climate** | **Inner Setting** |
|  | Communication within the hospital is effective. | **Networks & Communication** | **Inner Setting** |
|  | Clinical innovation and creativity to improve patient care are rewarded in my hospital. | **Learning Climate** | **Inner Setting** |
|  | Staff at the hospital have a sense of personal responsibility for improving patient care and outcomes. | **Culture** | **Inner Setting** |
|  | Staff at the hospital cooperate to maintain and improve effectiveness of patient care. | **Culture** | **Inner Setting** |
|  | Staff at the hospital are receptive to change in clinical processes. | **Culture** | **Inner Setting** |
|  | Clinical leadership at the hospital provides staff with information on hospital performance measures and guidelines. | **Reflecting & Evaluating** | **Process** |
|  | Clinical leadership at the hospital establishes clear goals for patient care processes and outcomes. | **Reflecting & Evaluating** | **Process** |
|  | Clinical leadership at the hospital provides staff with feedback/data on effects of clinical decisions. | **Reflecting & Evaluating** | **Process** |
|  | Clinical leadership at the hospital hold staff accountable for achieving results. | **Reflecting & Evaluating** | **Process** |
| **Demographic/**  **Personal Questions** | Are you trained as a: 1) pharmacist; 2) physician |  |  |
|  | How long have you worked at this hospital? |  |  |
|  | In what year were you first licensed as a physician/pharmacist? |  |  |
|  | Where did you attend pharmacy or medical school? |  |  |
|  | If applicable, where did you complete residency training? |  |  |
|  | If applicable, did you receive specialized Infectious Disease or Antibiotic Training? (y/n) |  |  |
|  | People sometimes volunteer to be the Antibiotic Steward within a hospital, and other times they are assigned by hospital management. Which answer best fits how you think it worked in your setting? |  |  |
|  | On a scale of 1 (very low) to 5 (very high), when you first started this antibiotic stewardship position, how much interest did you have in antibiotic stewardship?  *(Place a mark on the scale above)* |  |  |
|  | What is your involvement in the ASP?  - I’m the primary leader  - I co-lead the ASP with [open response]  - I do a lot of the work, but I’m not in charge  - Other, please reply: [open response] |  |  |
|  | Would you be willing to have us contact you to discuss these topics more? If so, please leave your name, and phone number and/or email address. |  |  |
|  | Name |  |  |
|  | Phone and/or email address |  |  |
|  | if you do NOT want to be contacted in the future, please indicate by selecting “Do not Contact.” |  |  |


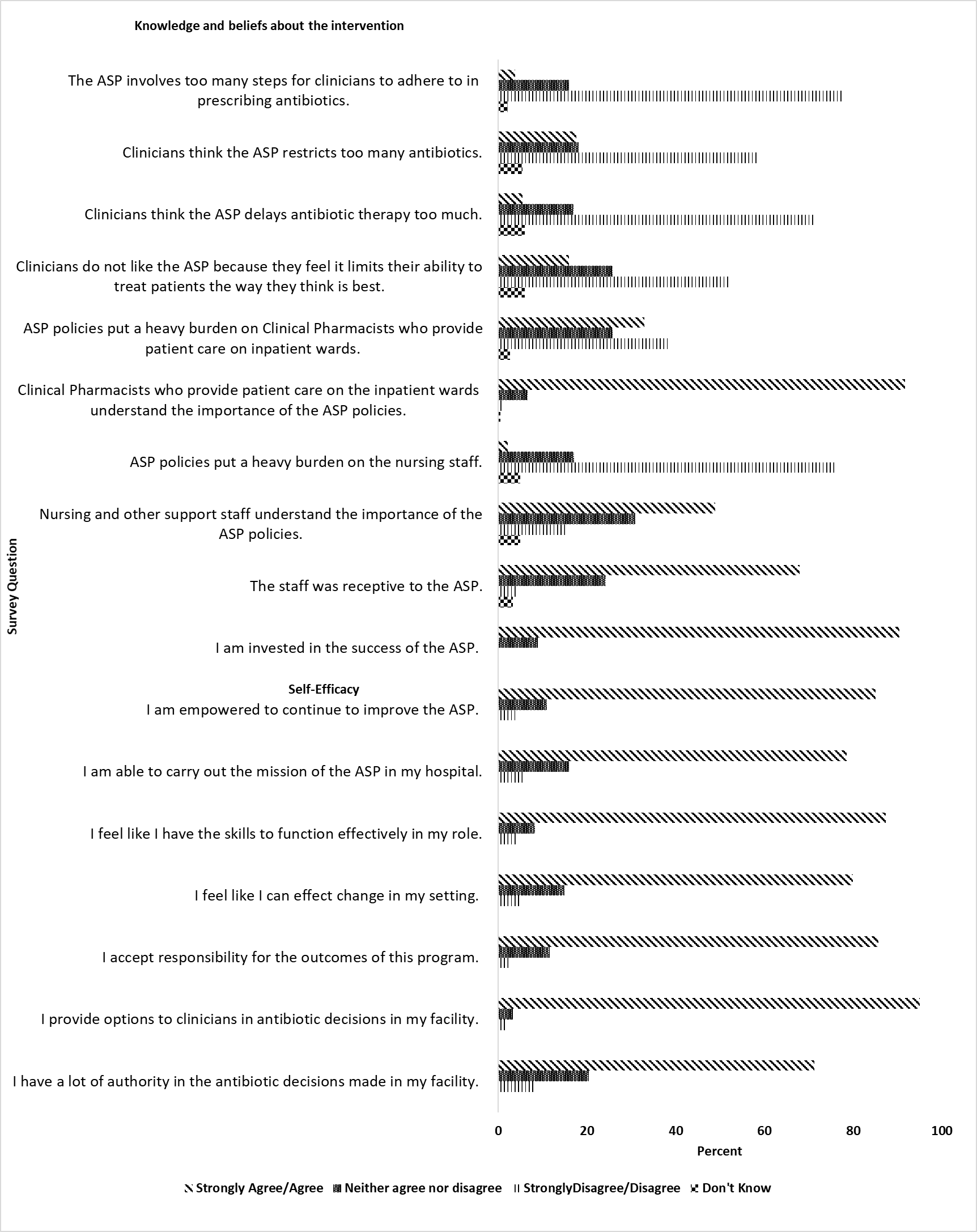

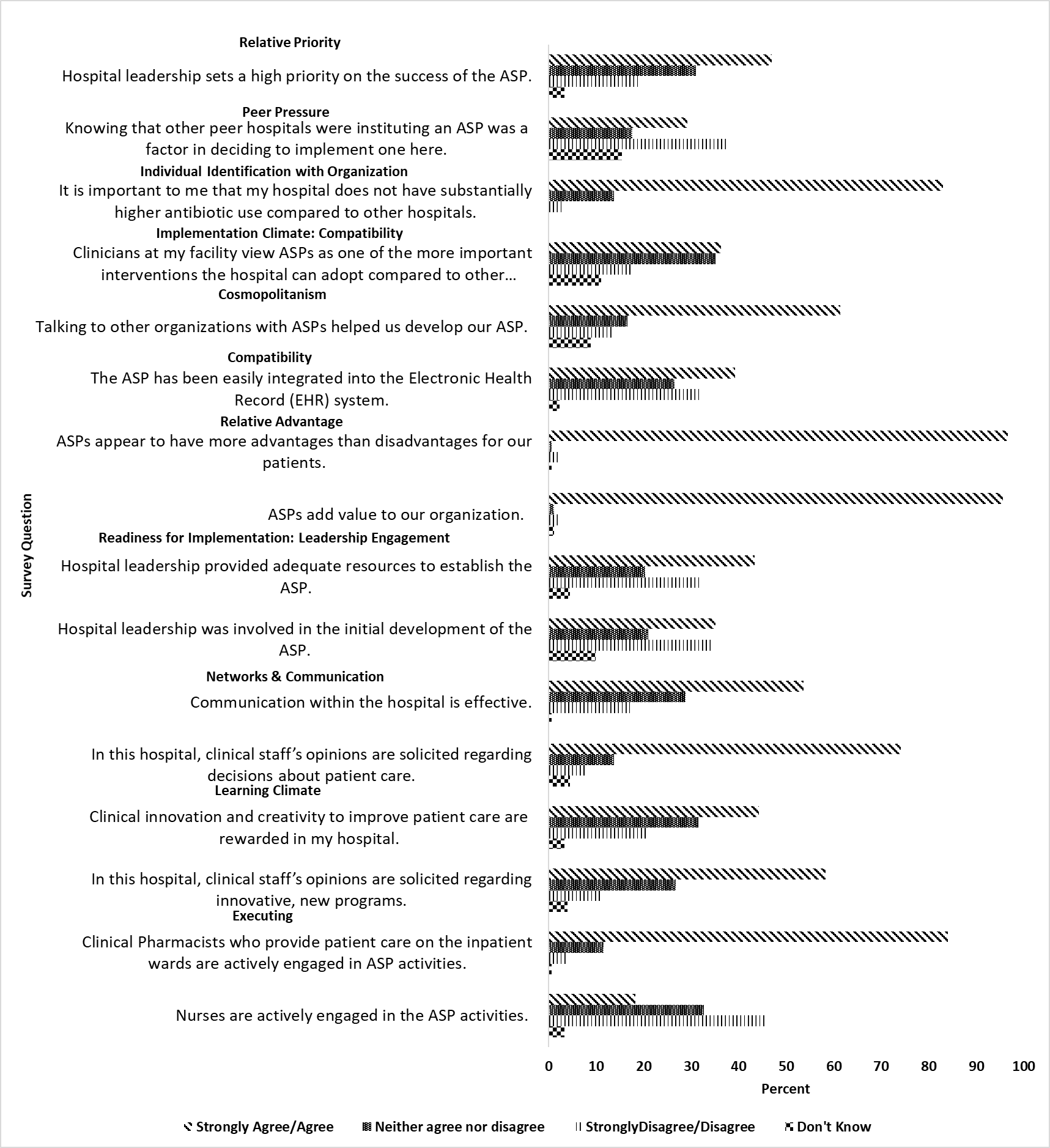


**b**

**a**

**Supplementary Figure 1: Distribution of responses to all survey questions**


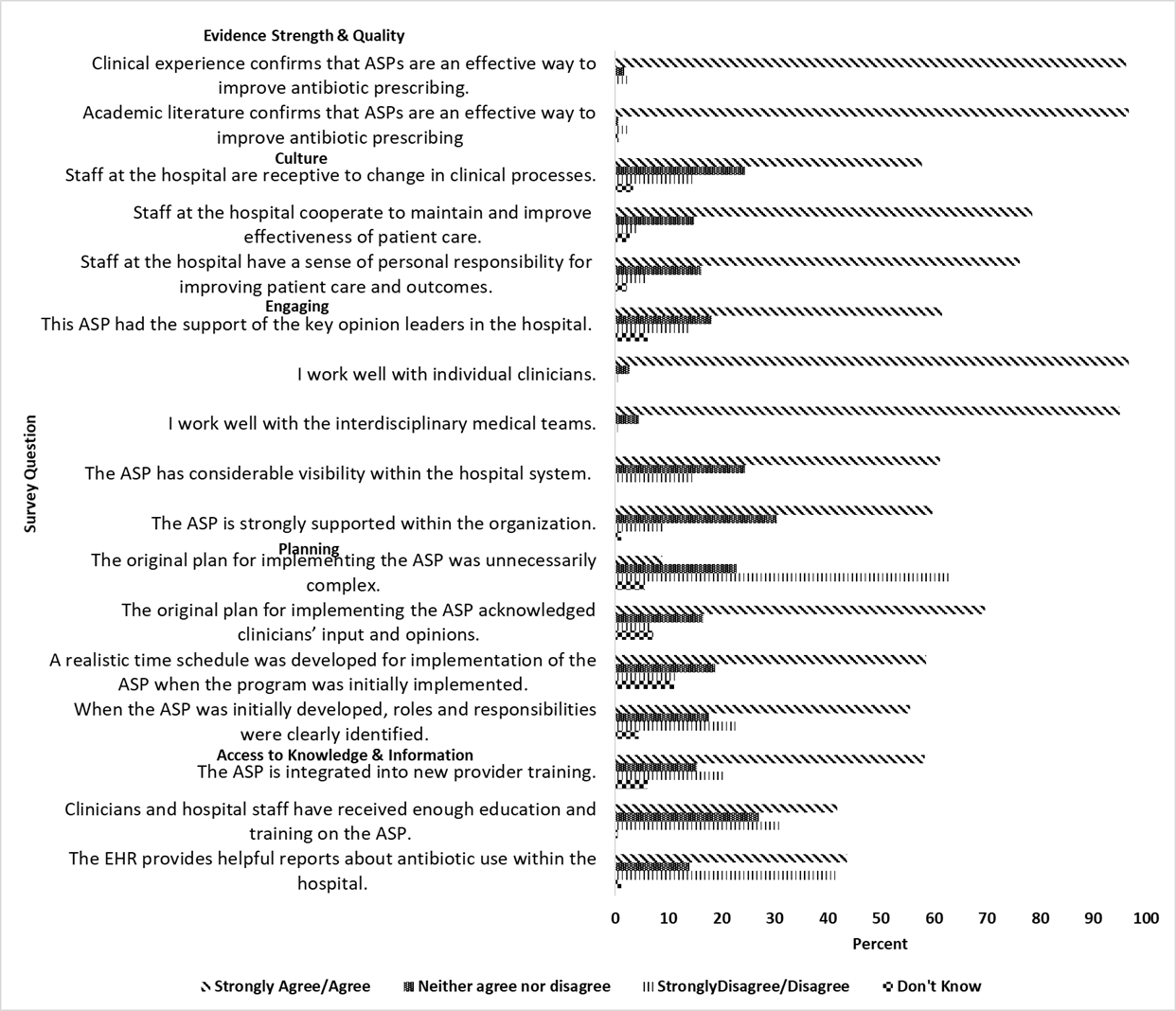

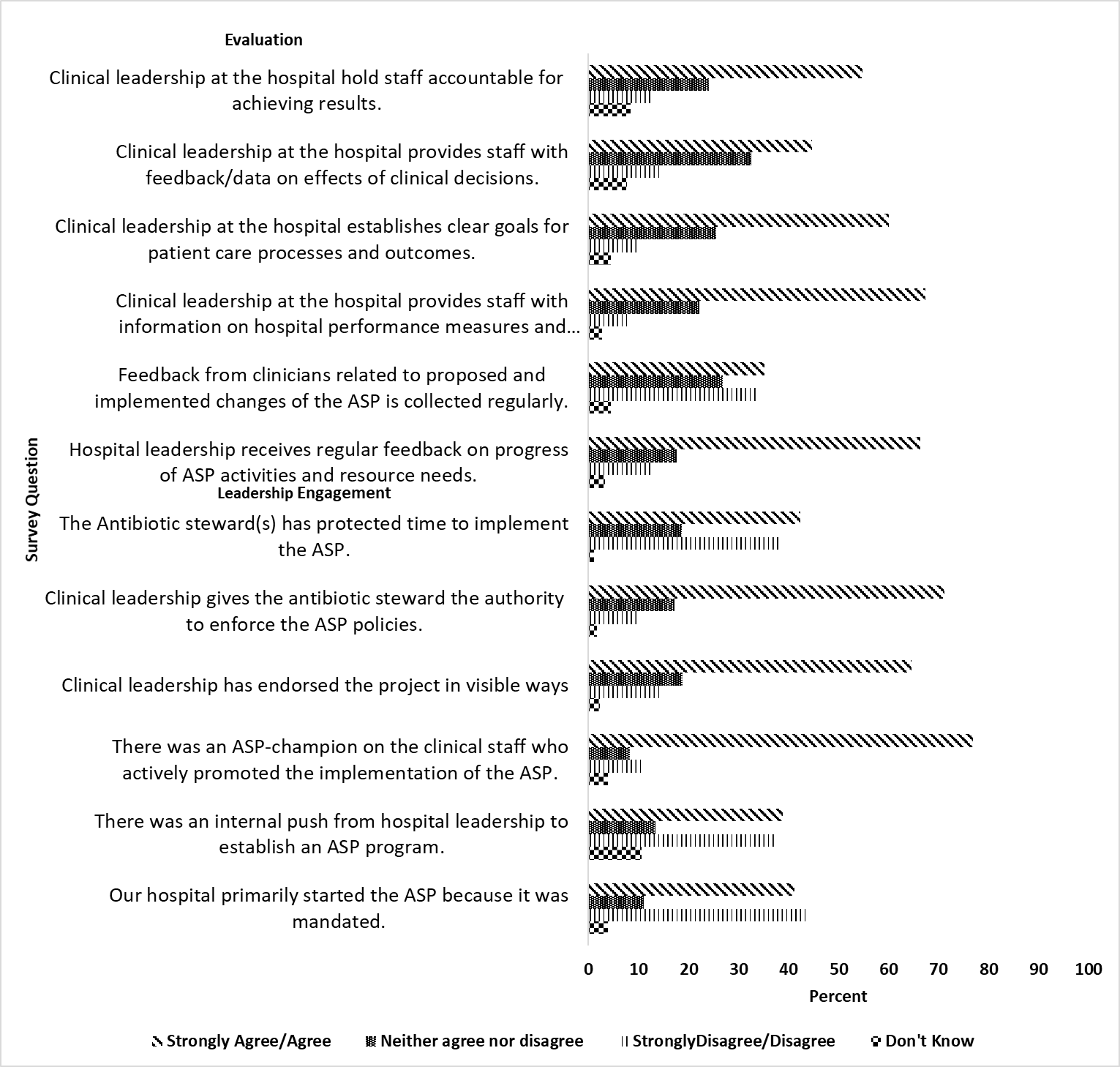


**d**

**c**
